# Survey on patients with undiagnosed diseases in Japan: potential patient numbers benefiting from Japan’s initiative on rare and undiagnosed diseases (IRUD)

**DOI:** 10.1186/s13023-018-0943-y

**Published:** 2018-11-20

**Authors:** Takeya Adachi, Noriaki Imanishi, Yasushi Ogawa, Yoshihiko Furusawa, Yoshihiko Izumida, Yoko Izumi, Makoto Suematsu

**Affiliations:** Japan Agency for Medical Research and Development (AMED), 22F Yomiuri Shimbun Bldg., 1-7-1 Otemachi, Chiyoda-ku, Tokyo 100-0004 Japan

**Keywords:** Survey, Japan, Initiative on rare and undiagnosed diseases (IRUD), Nan-byo, Rare diseases, Undiagnosed diseases, Undiagnosed disease program (UDP)

## Abstract

**Background:**

There is now an international partnership to establish global programs for patients with rare and undiagnosed diseases, involving interdisciplinary expert panels and phenotype-driven genetic analyses utilizing next-generation sequencing and analytics. Whereas it is crucial to have data such as the actual number of undiagnosed patients, to help inform the implementation plan with such programs, there have been no systematic studies to quantitate the numbers of patients principally because of the inherent difficulty in most health systems to identify patients whose condition has not yet been diagnosed and coded. Our national experience with a rare disease program, *Nan-Byo* which was established in 1972, and the more recently expanded Initiative on Rare and Undiagnosed Diseases (IRUD), provided a unique opportunity to design a cross-sectional study to ascertain the undiagnosed patients in Japan based on the IRUD referral criteria.

**Results:**

Two rounds of online surveys were performed: one survey targeting physicians affiliated with general hospitals (GH) and family clinics (FC) (the response rate: 30.6% (242/792)) and one nationwide survey targeting university hospitals (UH) in Japan (47.1% (839/1781)). A high percentage of doctors needing IRUD was seen in pediatrics at GH, FC, while there was a clear demand for IRUD in most departments at UH. We calculated the number of undiagnosed patients in Japan, as the “percentage of doctors needing IRUD” × “number of patients who would be referred to IRUD per doctor needing IRUD (cases/person)” × “total number of doctors in the relevant facilities in Japan (persons)”, resulting in 3681 cases in pediatrics/pediatric surgery and 33,703 cases in other departments, for a total of 37,384 cases.

**Conclusions:**

Our study revealed the extant demand for IRUD in most departments and 37,000+ potential patients with undiagnosed diseases in the Japanese health system. These data inform the establishment of an equitable, sustainable, efficient and effective outpatient-based IRUD. These findings would serve as a valuable reference for undiagnosed diseases programs in different international jurisdictions and for countries and regions who also share vision(s) for societal implementation that help to advance international efforts to support patients with rare diseases who are direly waiting for diagnosis, subsequent treatment and care.

## Background

There is an international partnership to establish global programs for patients with undiagnosed diseases in order to bring their “diagnostic odyssey” to an end—to finally help those patients who have been left in limbo without definitive diagnosis of their illness [1–6]. Such programs involve a combination of deep phenotyping of the patients by interdisciplinary expert panels, exhaustive genetic analysis by utilizing phenotype-driven next-generation sequencing and clinical and genomic data sharing. Outcomes from undiagnosed disease programs have proven highly successful in providing a confirmed diagnosis to many, varying between 25 and 60% depending on the program [7], with outcomes such as refined treatment and informing best care. To be equitable and sustainable, these programs must be planned with a clear vision of the implementation phase because it is challenging to maintain a diagnostic system comprising interdisciplinary specialists, counsellors and program coordinators. It is also imperative at the beginning to understand the numbers of people who may be referred to and accepted into the program to ensure any system costs, such as whole-exome and genome sequencing and subsequent analyses can be budgeted; for while these costs are gradually decreasing, they nevertheless need to be estimated as part of the business case for the program.

In 1972, a guideline of countermeasures against specifically defined rare diseases, called *Nan-Byo* in Japanese (literally “difficult” + “illness”), was established in Japan [8]. Since then, patients’ medical fees have been subsidized [9] and research has been promoted on *Nan-Byo* that meets the following four criteria: rarity, unknown etiology, no established treatment, and need for long-term care [8, 10]. However, patients whose conditions could not be diagnosed under *Nan-Byo* have been unable to receive the full benefit of our high-quality health systems and continued to experience a very difficult journey without a confirmed diagnosis. To rectify this situation, the Japan Agency for Medical Research and Development (AMED) launched the Initiative on Rare and Undiagnosed Diseases (IRUD). With this program, AMED strives to ensure diagnosis for patients whose conditions are hard to diagnose by standard medical practices due to extreme rarity or unprecedentedness, and to promote related research [6]. The IRUD has been designed to enable primary care physicians and general practitioners, whom such patients normally consult, to collaborate with doctors who are highly specialized in rare and intractable diseases. Together, they build a cohesive interdisciplinary informed phenotype. The deep phenotyping, combined with the power of derived from integrating the expertise of various intractable disease specialists and the results of cutting-edge genetic analysis is proving highly successful in arriving at a definitive diagnosis for many patients.

Many of the world’s leading undiagnosed disease programs are inpatient- or hospital-based [1]. However, because costs for hospitalization are limited and regulated strictly under the Diagnosis Procedure Combination policy [11], it was determined that in Japan an outpatient-based system would allow for a more effective use of various analyses that are covered by the health insurance system. To ensure equitable access in a system where patients with various symptoms may be required to make several outpatient clinic visits, it was decided to establish a network of easily accessible “base” hospitals, IRUD Clinical Centers, across the entire country. Consequently, it was a critical success element for the IRUD Clinical Centers to have information on resource needs informed by the potential scale of undiagnosed patient population. However, the difficulty in obtaining information on patients who are not yet diagnosed has prevented detailed studies on the number and diagnostic status of patients with undiagnosed diseases in Japan and in other countries, although there are reports from Australian and British groups that focus on rare diseases that have helped to inform estimates of the need [12, 13].

Given the importance of better understanding of current undiagnosed patients status for the strategic promotion of undiagnosed disease programs including the outpatient-based IRUD system in Japan, and to help contribute to global efforts to help patients with rare and other diseases that are difficult to diagnose [14–16], we designed a cross-sectional study to ascertain the actual number of patients with undiagnosed diseases in Japan. This survey targeting all national and public university hospitals (NUH), private university hospitals (PUH; main and branch locations: PUH-main and PUH-branch, respectively), general hospitals (GH), and family clinics (FC) in Japan, suggested that there are a wide-range of demand for IRUD and at least 37,000 potential patients with undiagnosed diseases in Japan. It is hoped that this data would also contribute to international efforts that aim to establish and implement undiagnosed disease programs in their jurisdiction.

## Methods

### Survey instruments

This study design consisted of two surveys. Survey-1 was carried out online in February 2016, targeting physicians in the Greater Tokyo Area and the Kinki region. Survey-2 was conducted online from November 2016 to January 2017 with doctors from all over Japan who worked at NUH (55 facilities), PUH-main (29 facilities), and PUH-branch (53 facilities, twelve clinics were excluded from this study).

The targets of the survey were physicians from pediatrics/pediatric surgery and from other departments (including general internal medicine/family medicine, pulmonology, cardiology, gastroenterology, nephrology, neurology, endocrinology, osteopathy, hematology, dermatology, allergology, and rheumatology). While the pediatrics/pediatric surgery departments had proportionately fewer departments and fewer overall patient numbers it was expected to have more opportunities to examine patients with genetic disorders. Thus, as predicted in the survey design phase, approximately one-third of cases were collected from pediatrics/pediatric surgery department.

**Fig. 1:**
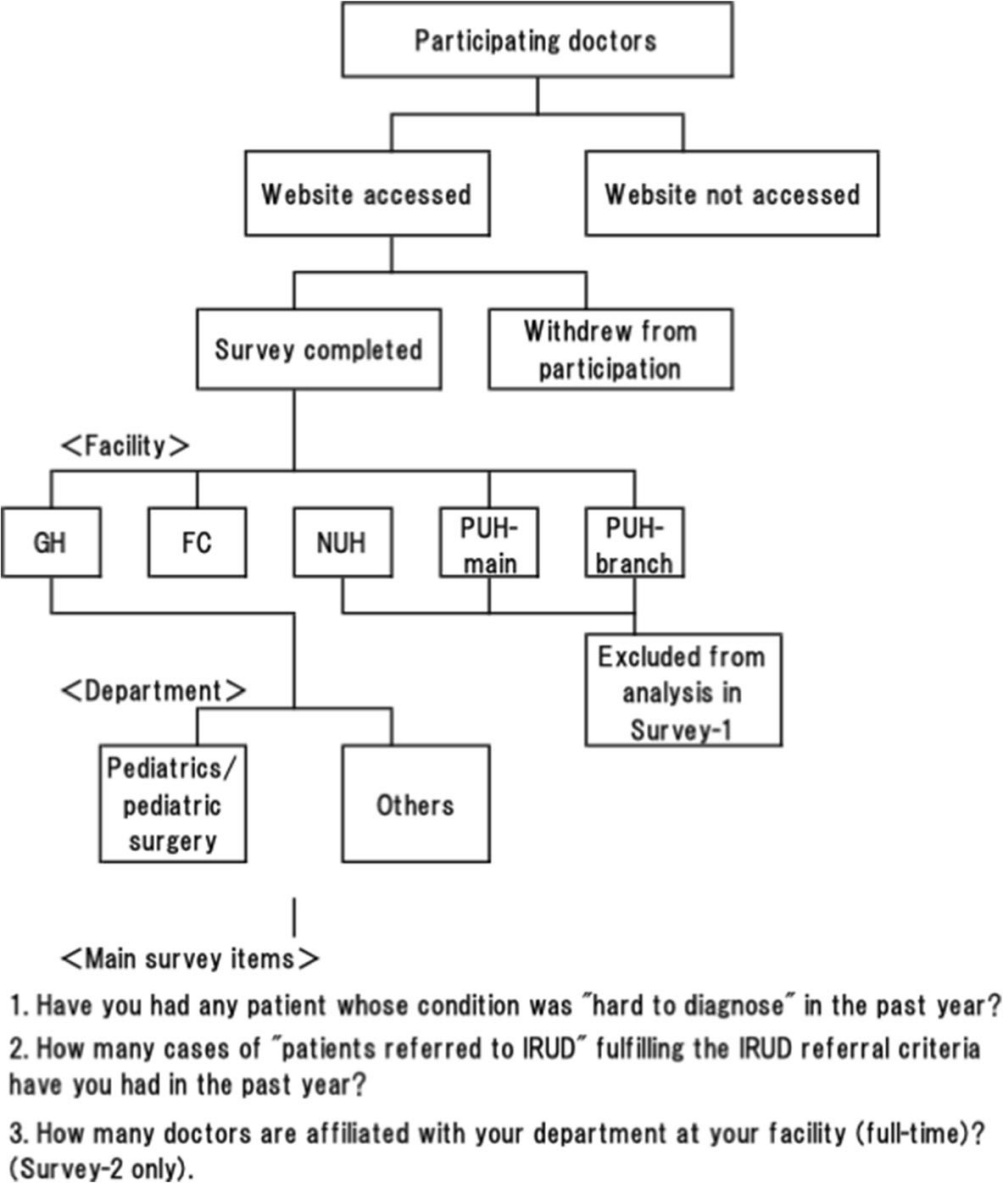
Schematic diagram of the survey instrument for Survey-1. The physicians can consider referring each difficult case when the following criteria are met: 1. The patient remains undiagnosed for six months or longer (not necessary for infants) and the symptom(s) affects his/her daily life; AND, 2–1. There exists an objective sign(s) that cannot be reduced to a single organ; OR 2–2. There exists direct or indirect evidence of a genetic etiology as likely (e.g., similar symptom(s) found in the patient’s relatives)

Survey-1 was carried out with a panel of 982 doctors from the Greater Tokyo Area and the Kinki region who participated through the online survey system by a survey company (Medical Collective Intelligence Co., Ltd., Tokyo, Japan). The URL of the survey was electronically distributed during the survey period (by e-mail only). The access log of the website was checked, and 538 doctors who did not access the website and 144 doctors who withdrew from participation after accessing the website were excluded as “doctors with no interest in undiagnosed diseases”. Because insufficient responses were collected from university hospitals (NUH: 13; PUH: 45 vs. GH: 132; FC: 110), only GH and FC (total of 792 participating doctors) were analyzed in Survey-1 (Fig. 1). Before starting the survey, information about IRUD concept was provided to participants for the better understanding of each question [6]. Following eligible criteria of IRUD were also shown: 1. The patient remains undiagnosed for six months or longer (not necessary for infants) and the symptom(s) affects his/her daily life; AND, 2–1. There exists an objective sign(s) that cannot be reduced to a single organ; OR 2–2. There exists direct or indirect evidence of a genetic etiology as likely (e.g., similar symptom(s) found in the patient’s relatives) [6]. Those who completed the survey before the deadline (242 in total; pediatrics/pediatric surgery: 81; others: 161; response rate 30.6% (242/792)) were categorized by facility and department, then analyzed for the main survey items (Fig. 1).

Survey-2 targeted standard doctors via the director of each NUH and PUH, because not enough responses to Survey-1 came from these institutions. In principle, three representative doctors from the pediatrics/pediatric surgery departments and 10 from other departments who were full-time society-certified physicians (assistant/associate professors and lecturers, etc.) and who graduated from medical school at least 10 years earlier were asked to participate. Of the 1781 preregistered doctors, 839 doctors participated and completed the survey with the online survey system (response rate: 47.1%).

### Subjects

Of the 242 doctors who completed Survey-1 (except those from university hospitals), 132 were from GH (pediatrics/pediatric surgery: 45; others: 87), 110 from FC (pediatrics/pediatric surgery: 36; others: 74) (Table 1). Of the 839 doctors who completed Survey-2, 355 were from NUH (pediatrics/pediatric surgery: 75; others: 280), 285 from PUH-main (pediatrics/pediatric surgery: 40; others: 245), and 199 from PUH-branch (pediatrics/pediatric surgery: 33; others: 166) (Table 2).

**Table 1:** Characteristics of the survey response groups for Survey-1

Survey-1	General Hospital (GH)				Family Clinics (FC)			
	Pediatrics/pediatric surgery		Others		Pediatrics/pediatric surgery		Others	
No. of doctors who completed the survey	45		87		36		74	
Sex								
Male	39	86.7%	80	92.0%	33	91.7%	64	86.5%
Female	6	13.3%	7	8.0%	3	8.3%	10	13.5%
Age								
20s	0	0.0%	1	1.1%	0	0.0%	0	0.0%
30s	10	22.2%	12	13.8%	1	2.8%	5	6.8%
40s	8	17.8%	33	37.9%	5	13.9%	23	31.1%
50s	18	40.0%	35	40.2%	19	52.8%	34	45.9%
60+	9	20.0%	6	6.9%	11	30.6%	12	16.2%
Years since obtaining medical license								
Under 10 years	2	4.4%	3	3.4%	0	0.0%	2	2.7%
10–14	8	17.8%	10	11.5%	0	0.0%	6	8.1%
15–19	3	6.7%	20	23.0%	2	5.6%	8	10.8%
20–24	9	20.0%	20	23.0%	7	19.4%	17	23.0%
25–29	8	17.8%	23	26.4%	12	33.3%	19	25.7%
30+	15	33.3%	11	12.6%	15	41.7%	22	29.7%
No. of outpatients in the past month								
Under 100 cases	6	13.3%	15	17.2%	0	0.0%	2	2.7%
100–199 cases	14	31.1%	15	17.2%	2	5.6%	6	8.1%
200–299 cases	9	20.0%	24	27.6%	4	11.1%	4	5.4%
300–399 cases	6	13.3%	10	11.5%	0	0.0%	7	9.5%
400–599 cases	7	20.0%	15	17.2%	6	16.7%	17	23.0%
600–799 cases	1	0.0%	2	2.3%	12	33.3%	13	17.6%
800–999 cases	0	0.0%	4	4.6%	5	13.9%	9	12.2%
1000+ cases	2	4.4%	2	2.3%	7	19.4%	16	21.6%

**Table 2:** Characteristics of the survey response groups for Survey-2

Survey-2	National/public university hospitals (NUH)				Private university hospitals (main) (PUH-main)				Private university hospitals (branch) (PUH-branch)			
	Pediatrics/pediatric surgery		Others		Pediatrics/pediatric surgery		Others		Pediatrics/pediatric surgery		Others	
No. of doctors who completed the survey	75		280		40		245		33		166	
Sex												
Male	63	84.0%	257	91.8%	31	77.5%	215	87.8%	28	84.8%	149	89.8%
Female	12	16.0%	23	8.2%	9	22.5%	30	12.2%	5	15.2%	17	10.2%
Age												
20s	0	0.0%	0	0.0%	0	0.0%	0	0.0%	0	0.0%	0	0.0%
30s	23	30.7%	62	22.1%	7	17.5%	59	24.1%	6	18.2%	26	15.7%
40s	39	52.0%	162	57.9%	18	45.0%	114	46.5%	12	36.4%	70	42.2%
50s	10	13.3%	53	18.9%	10	25.0%	57	23.3%	13	39.4%	57	34.3%
60+	3	4.0%	3	1.1%	5	12.5%	15	6.1%	2	6.1%	13	7.8%
Years since obtaining medical license												
Under 10 years	0	0.0%	4	1.4%	1	2.5%	12	4.9%	1	3.0%	5	3.0%
10–14	27	36.0%	61	21.8%	3	7.5%	56	22.9%	6	18.2%	31	18.7%
15–19	24	32.0%	105	37.5%	13	32.5%	59	24.1%	7	21.2%	35	21.1%
20–24	16	21.3%	63	22.5%	11	27.5%	52	21.2%	5	15.2%	33	19.9%
25–29	3	4.0%	27	9.6%	5	12.5%	28	11.4%	3	9.1%	34	20.5%
30+	5	6.7%	20	7.1%	7	17.5%	38	15.5%	11	33.3%	28	16.9%
No. of outpatients in the past month												
Under 100 cases	38	50.7%	86	30.7%	13	32.5%	61	24.9%	5	15.2%	23	13.9%
100–199 cases	29	38.7%	110	39.3%	15	37.5%	75	30.6%	9	27.3%	52	31.3%
200–299 cases	8	10.7%	51	18.2%	9	22.5%	67	27.3%	11	33.3%	37	22.3%
300–399 cases	0	0.0%	19	6.8%	2	5.0%	20	8.2%	5	15.2%	22	13.3%
400–599 cases	0	0.0%	9	3.2%	1	2.5%	19	7.8%	2	6.1%	19	11.4%
600–799 cases	0	0.0%	2	0.7%	0	0.0%	3	1.2%	1	3.0%	10	6.0%
800–999 cases	0	0.0%	1	0.4%	0	0.0%	0	0.0%	0	0.0%	1	0.6%
1000+ cases	0	0.0%	2	0.7%	0	0.0%	0	0.0%	0	0.0%	2	1.2%

### Questionnaire

Our study targeted doctors in Japan, to ascertain the “number of undiagnosed patients who would be referred to IRUD”. The main items of the surveys were as follows:

1. Have you had any patient whose condition was “hard to diagnose” in the past year?
2. How many cases of “patients referred to IRUD” fulfilling the IRUD referral criteria [6] have you had in the past year?
3. How many doctors are affiliated with your department at your facility (full-time)? (Survey-2 only)

### Analysis

Of the doctors who completed the survey, those who had a “patient whose condition is hard to diagnose” were termed “doctors needing IRUD”. The data were extrapolated to estimate the number of patients with undiagnosed diseases in the entire country (see below). Analyses were performed on pediatrics/pediatric surgery versus other departments in both surveys, and in Survey-2, we also analyzed the individual departments because of ample responses (Table 6).

• “Number of patients with undiagnosed diseases in Japan who would be referred to IRUD (cases)” = “percentage of doctors needing IRUD”* × “number of patients who would be referred to IRUD per doctor needing IRUD (cases/person; average value per doctor)”** × “total number of doctors in the relevant facilities in Japan (persons)”***.

* “Percentage of doctors needing IRUD” = number of doctors needing IRUD (persons)/number of participating doctors (persons).

** “Number of patients who would be referred to IRUD per doctor needing IRUD (cases/person; average value per doctor)” is derived from the second question of the questionnaire.

*** Survey-1: Total number of doctors in GH and FC (persons) = total number of doctors working for hospitals and clinics in the entire country [17] – total number of university hospital doctors [18]).

*** Survey-2: Total number of doctors in NUH, PUH-main, and PUH-branch (persons) = total number of doctors per department per facility (persons/facility) **** × number of relevant facilities in Japan × correction value*****.

**** Number of doctors per department per facility was calculated based on survey item 3 of Survey-2: “Total number of doctors affiliated with your department at your facility (full-time)”. Number of doctors per department was only analyzed individually if there were at least 20 valid responses. Departments with fewer than 20 responses were pooled as “others” (Table 6).

***** Total number of doctors in the relevant facilities calculated as sum of the number of doctors per department per facility (persons/facility) × number of relevant facilities in Japan was multiplied by 0.519 for NUH and 0.605 for PUH in order to match the statistical total number of doctors in Japan [19].

### Statistics

Statistical analyses were performed using GraphPad Prism 6.0. The results were expressed as the percentage or number of cases. The differences in “percentage of doctors needing IRUD” were assessed for significance using chi-squared analysis.

## Results

### Characteristics of the survey response groups

The ages of the doctors from GH and FC who completed Survey-1 ranged from 20 years to 60 years with a number of older participants. With the exception of FC pediatrics/pediatric surgery, a broad distribution was observed for the number of years since obtaining their license, ranging from under 10 years to approximately 35 years; over half of the doctors had been licensed for at least 20 years. In GH, most doctors had 100 to 300 outpatient visits in the past month, while the majority in FC had over 600 cases (Table 1). The peak age range of NUH, PUH-main, and PUH-branch doctors in Survey-2 was the 40s. This is probably due to the participants being specified as “full-time society-certified physicians” (assistant/associate professors and lecturers, etc.) who graduated from medical school at least 10 years earlier. The peak range of the number of years since obtaining medical license was 10 to 19 years for NUH and PUH-main. For PUH-branch, a broader range of 10 to 29 years was observed, which was similar in trend to GH and FC. Approximately 90% of the doctors in NUH and PUH-main had fewer than 300 outpatient visits in the previous month, while the range of the number of outpatient visits that the doctors in PUH-branch received in the same period was relatively wide (Table 2), compared with that of GH in Survey-1.

### Demand for IRUD in Japan

In Survey-1, whereas a significant demand for IRUD was shown in every department, the “percentage of doctors needing IRUD” in GH and FC was higher in pediatrics/pediatric surgery than in other departments (GH: 36.8% vs. 19.4%, *p* < 0.001; FC: 32.9% vs. 18.2%, *p* < 0.01) (Table 3). Similarly, there was a high demand for IRUD in pediatrics/pediatric surgery of PUH-main (90.0% vs. 69.4%, p < 0.01), while little difference was found in NUH between pediatrics/pediatric surgery and other departments (88.0% vs. 84.6%) (Table 4). Regarding PUH-branch, no significant difference was found (66.7% vs. 75.9%), although there was a trend of higher demand for other departments (Table 4).

**Table 3:** Number of patients with undiagnosed diseases in Japanese general hospitals and family clinics who would be referred to IRUD

Survey-1	Pediatrics/pediatric surgery		Other departments	
	General hospitals (GH)	Family clinics (FC)	General hospitals (GH)	Family clinics (FC)
Percentage of doctors needing IRUD	36.8% (35/95)	32.9% (25/76)	19.4% (65/335)	18.2% (52/286)
Number of participating doctors (persons)	95	76	335	286
Number of doctors completed the survey (persons)	45	36	87	74
Number of doctors needing IRUD (persons)	35	25	65	52
Number of patients who would be referred to IRUD per doctor needing IRUD (cases/person)	0.3	0.1	0.6	0.1
Total number of doctors in the relevant facilities in Japan (persons)	9192	6677	155,907	95,207
Number of patients with undiagnosed diseases in Japan who would be referred to IRUD (cases)	1063	264	18,133	2337

**Table 4:** Number of patients with undiagnosed diseases in Japanese university hospitals who would be referred to IRUD

Survey-2	Pediatrics/pediatric surgery			Other departments		
	NUH	PUH-main	PUH-branch	NUH	PUH-main	PUH-branch
Percentage of doctors needing IRUD	88.0% (66/75)	90.0% (36/40)	66.7% (22/33)	84.6% (237/280)	69.4% (170/245)	75.9% (126/166)
Number of participating doctors (persons)^a^	75	40	33	280	245	166
Number of doctors needing IRUD (persons)	66	36	22	237	170	126
Number of patients who would be referred to IRUD per doctor needing IRUD (cases/person)	2.4	1.2	0.9	0.8	0.4	0.3
Total number of doctors in the relevant facilities in Japan (persons)	692	581	408	12,320	10,474	6955
Number of patients with undiagnosed diseases in Japan who would be referred to IRUD (cases)	1462	652	240	8338	3098	1797

^a^In Survery-2, all the participating doctors completed the survey

There were fewer cases of patients who would be referred to IRUD per doctor needing IRUD in FC than in GH, but there were 0.1 cases/doctor of patients who would be referred to IRUD in both groups of departments at FC (Table 3). This number was 2.4 and 0.8 for pediatrics/pediatric surgery and other departments, respectively, at NUH; 1.2 and 0.4 for PUH-main; and 0.9 and 0.3 at PUH-branch (Table 4). There appeared to be relatively high patient demand in pediatrics/pediatric surgery at NUH and PUH-main.

### Number of patients with undiagnosed diseases in Japan who would be referred to IRUD

From the above, we found that there were 1462 and 8338 cases of patients with undiagnosed diseases in Japan who would be referred to IRUD from the NUH pediatrics/pediatric surgery departments and other departments, respectively (Table 4). For PUH-main, there were 652 and 3098 cases in pediatrics/pediatric surgery and other departments, respectively, and for PUH-branch, there were 240 and 1797 cases, respectively (Table 4). For GH, there were 1063 cases in pediatrics/pediatric surgery and 18,133 cases in other departments, and for FC, there were 264 and 2337 cases, respectively (Table 3). These results show that demand for IRUD exists not only in university hospitals but also in regional GH and FC.

Table 5 summarizes Survey-1 and Survey-2. Together, there were 3681 cases of patients with undiagnosed diseases in Japan who would be referred to IRUD from the pediatrics/pediatric surgery departments of NUH, PUH, GH, and FC, and 33,703 cases from other departments. This total of 37,384 cases suggests that there are potentially many patients with undiagnosed diseases in Japan.

**Table 5:** Number of patients introduced to the IRUD diagnosis network in Japan

Survey-1 + Survey-2	Number of patients with undiagnosed diseases in Japan who would be referred to IRUD (cases)		
	Pediatrics/pediatric surgery	Other departments	Total
General hospitals (GH)	1063	18,133	19,196
Family clinics (FC)	264	2337	2601
National/public university hospitals (NUH)	1462	8338	9800
Private university hospitals (main) (PUH-main)	652	3098	3750
Private university hospitals (branch) (PUH-branch)	240	1797	2037
Total	3681	33,703	37,384

### Patients with undiagnosed diseases per university hospital department

Table 6 shows the department-by-department breakdown of Survey-2 responses. Departments with valid responses from at least 20 people were analyzed individually. All departments with less than 20 responses were pooled as “others”. Based on the “percentage of doctors needing IRUD”, neurology had the largest number of doctors who wished to refer their patients to IRUD, followed by general internal/family medicine, dermatology, and rheumatology. Based on the number of patients who would be referred to IRUD per doctor needing IRUD, there were many patients with undiagnosed diseases in pediatrics, followed by neurology, nephrology, and otorhinolaryngology, specifically 2.1, 1.5, 0.9, and 0.8 patients per doctor, respectively. Extrapolated to a national scale, these numbers became 2306, 1123, 461, and 771 patients, respectively, indicating a relatively high demand for IRUD from patients with undiagnosed diseases.

**Table 6:** Number of patients with undiagnosed diseases per university hospital department

Survey-2	Department	Percentage of doctors needing IRUD	Number of participating doctors (persons)	Number of patients who would be referred to IRUD per doctor needing IRUD (cases/person)	Total number of doctors per department (persons)	Number of patients with undiagnosed diseases in Japan who would be referred to IRUD (cases)
Pediatrics/pediatric surgery	Pediatrics	85.6%	125	2.1	1272	2306
	Pediatric surgery	73.9%	23	0.3	409	82
Other departments	Neurology	100.0%	47	1.5	741	1123
	General internal/family medicine	100.0%	29	0.7	625	453
	Dermatology	96.4%	55	0.6	786	425
	Rheumatology	93.5%	46	0.7	786	523
	Otorhinolaryngology	90.5%	21	0.8	1079	771
	Gastroenterology	85.0%	60	0.3	1532	403
	Pulmonology	84.1%	44	0.4	812	258
	Orthopedics	80.0%	45	0.2	1329	266
	Hematology	73.0%	37	0.7	623	301
	Cardiology	71.9%	64	0.5	1170	457
	Diabetes and metabolism	71.1%	45	0.6	858	362
	Nephrology	68.4%	38	0.9	770	461
	Gastrointestinal surgery	56.5%	23	0.1	1485	65
	Others	59.1%	137	0.2	17,153	2106

## Discussion

The IRUD was launched in 2015 as an outpatient-based program for patients with undiagnosed diseases, with a vision for nationwide implementation in the future. To expand equitably and efficiently to a national scale, analysis of the actual status of patients with undiagnosed diseases in Japan has provided important and fundamental data for ensuring sustainability of the program. In this study, doctors who had “patients whose condition is difficult to diagnose” in the past year were defined as “doctors needing IRUD”, and the number of patients with undiagnosed diseases in Japan was estimated based on the rate of such doctors and the number of patients who would be referred to IRUD by these doctors. In other words, this is a cross-sectional study on the number of patients with undiagnosed diseases in Japan based on the IRUD referral criteria.

Survey-1 targeted a panel of doctors from the Greater Tokyo Area and the Kinki region who participated through a survey company. We obtained sufficient responses at GH and FC by using an online survey. Of the participating doctors, those who did not access the website or who did but withdrew from participation were excluded from the study as “doctors with no interest in undiagnosed diseases”; however, it cannot be ruled out that some of them actually might be “doctors needing IRUD”. Additionally, there are many university hospitals in the targeted Greater Tokyo Area and the Kinki region. Patients from these regions with a condition that is relatively hard to diagnose might already have been referred to a university hospital, resulting in a smaller number of “patients who would be referred to IRUD” here than elsewhere. For these reasons, the “number of patients with undiagnosed diseases in Japan who would be referred to IRUD” in Survey-1 might actually be an underestimation. On the other hand, the levels of workup at GH and FC are not equivalent to those at university hospitals, which might link to the overestimation of the undiagnosed patients in non-university hospitals.

Moreover, our estimates of patients with undiagnosed diseases in Japan who would be referred to IRUD indicate that there is more demand for IRUD in other departments (33,703 cases) than in pediatrics/pediatric surgery (3681 cases). The biggest reason for this difference is the smaller number of pediatricians/pediatric surgeons in Japan (17,550) compared with all other doctors (280,863). In fact, the “percentage of doctors needing IRUD” shows that there is more demand for IRUD in pediatrics/pediatric surgery than in other departments (58.3% vs. 49.5%, *p* < 0.01), which supports the fact that pediatrics/pediatric surgery has the highest “number of patients who would be referred to IRUD per doctor needing IRUD” among all departments. One possible cause of this apparent discrepancy is an IRUD referral criterion used in this study, namely, “there exists an objective sign(s) that cannot be reduced to a single organ” [6]. Such patients may be consulting multiple specialists, resulting in the overestimation in number of patients in other departments. In addition, there may actually be more IRUD demand in pediatrics/pediatric surgery than our results indicate, because pediatric patients are also treated at “other departments”, such as dermatology and otorhinolaryngology. The relatively small “number of patients who would be referred to IRUD per doctor needing IRUD” of GH in pediatrics/pediatric surgery (0.6 cases/person), if compared to that of university hospitals (2.4, 1.2 and 0.9 for NUH, PUH-main, and PUH-branch, respectively), might be caused by smaller sample size of non-university population, which might contribute to its underestimation.

Prior to this study, interdisciplinary diagnosis committees have begun conferring at IRUD Clinical Centers. They confer once or twice a month at one of these centers, discussing two to four cases per conference. If approximately 100 cases were to be discussed per year (4 cases/conference × 2 times/month × 12 months/year = 96 cases/year), approximately 37 clinical centers for 10 years would be necessary for the minimal estimation of 37,384 potential cases in Japan (Table 5). Moreover, because there are more patients with undiagnosed diseases in regional GH and FC (21,797 cases in total) than in university hospitals (15,587 cases in total), it would be also desirable to assemble patients at regional collaborating hospitals. Based on these results, IRUD has been striving to set up 30 to 40 clinical centers across Japan, with 10 collaborating hospitals per clinical center. Currently (April 2018), 37 clinical centers and more than 370 collaborating hospitals have been set up and 3416 patients were referred to IRUD in the first 2 years [20]. A long-term study would be needed to determine the patient capacity of the current system.

Prior to the introduction of IRUD, patients with undiagnosed diseases have been unable to get a diagnosis, but they also do not know where to get the medical attention they need. They do not receive adequate assistance in covering medical costs and experience various symptoms. However, advances in domestic and international projects for undiagnosed diseases have contributed to the diagnosis of many such patients, also elucidating the pathology, treatment(s), and clinical care [7, 21, 22]. Because the term “undiagnosed disease” includes rare diseases with low incidence reports as well as novel diseases that have not been reported before, the International Rare Diseases Research Consortium (IRDiRC) has also launched activities to accelerate diagnosis as part of a 10-year strategy [14, 16], to which AMED has been contributing since the planning phase [23, 24]. An additional goal of this strategy is the development of a method to measure its own impact, that is, to determine whether patients are actually benefitting from the diagnosis and treatment modalities that the project has promoted. The number of potential patients with undiagnosed diseases may very well serve as an index of this goal.

Very little information has been available on the status of patients with undiagnosed diseases worldwide due to the difficulty of investigating patients whose condition has not been diagnosed. Our findings contribute to establishing an undiagnosed disease program in Japan and would also serve as valuable reference material in considering outpatient-based undiagnosed disease programs, as well as for advancing international efforts to support patients with rare and undiagnosed diseases.

## Conclusion

This survey aimed to ascertain the number of patients with undiagnosed diseases in Japan as a basis for establishing a research program on the IRUD diagnostic system. Overall, it suggests there are at least 37,000 patients with undiagnosed diseases in Japan, with 3681 cases in pediatrics/pediatric surgery and 33,703 cases in other departments. These findings contribute to the efficient and effective establishment of an undiagnosed disease program in Japan and provide globally significant data for countries that plan to launch their own outpatient-based undiagnosed disease program. Further utilization of our results is expected in the strategic implementation of undiagnosed disease programs and promotion of international efforts to help patients with rare and undiagnosed diseases.

## Abbreviations

AMED: Japan Agency for Medical Research and Development
FC: Family clinics
GH: General hospitals
IRDiRC: International Rare Diseases Research Consortium
IRUD: Initiative on Rare and Undiagnosed Diseases
NUH: National and public university hospitals
PUH: Private university hospitals
